# A Vision of Future Healthcare: Potential Opportunities and Risks of Systems Medicine from a Citizen and Patient Perspective—Results of a Qualitative Study

**DOI:** 10.3390/ijerph18189879

**Published:** 2021-09-19

**Authors:** Clarissa Lemmen, Dusan Simic, Stephanie Stock

**Affiliations:** Institute for Health Economics and Clinical Epidemiology (IGKE), University of Cologne, Faculty of Medicine and University Hospital Cologne, 50935 Cologne, Germany; dusan.simic@uk-koeln.de (D.S.); stephanie.stock@uk-koeln.de (S.S.)

**Keywords:** systems medicine, systems biology, big data, implementation, precision medicine, personalized medicine, artificial intelligence, digital health

## Abstract

Advances in (bio)medicine and technological innovations make it possible to combine high-dimensional, heterogeneous health data to better understand causes of diseases and make them usable for predictive, preventive, and precision medicine. This study aimed to determine views on and expectations of “systems medicine” from the perspective of citizens and patients in six focus group interviews, all transcribed verbatim and content analyzed. A future vision of the use of systems medicine in healthcare served as a stimulus for the discussion. The results show that although certain aspects of systems medicine were seen positive (e.g., use of smart technology, digitalization, and networking in healthcare), the perceived risks dominated. The high degree of technification was perceived as emotionally burdensome (e.g., reduction of people to their data, loss of control, dehumanization). The risk-benefit balance for the use of risk-prediction models for disease events and trajectories was rated as rather negative. There were normative and ethical concerns about unwanted data use, discrimination, and restriction of fundamental rights. These concerns and needs of citizens and patients must be addressed in policy frameworks and health policy implementation strategies to reduce negative emotions and attitudes toward systems medicine and to take advantage of its opportunities.

## 1. Introduction

For several years now, the concept of systems medicine has been discussed as a pioneering approach to healthcare. In this vision of the future, systems medicine promises to provide new impetus for participative, proactive, and preventive healthcare [1,2,3,4,5]. It is not yet clear how and in what period the systems medicine approach will find its way into the healthcare system. Due to the complexity and the necessary integration of heterogeneous concepts there is no consensual understanding of systems medicine so far [6,7]. This complicates a shared vision and the development of strategies for the implementation of systems medicine in healthcare [8].

Systems medicine is not a medical discipline in the traditional sense, but terms a relatively young interdisciplinary approach that brings together (bio)medical knowledge and digital technologies for systems-oriented thinking and action [9,10]. It uses and combines extensive molecular biological, clinical, and demographic data, including environmental factors, to evaluate complex biological relationships. This systematic integration of diverse data sources is intended to improve our understanding of the causes of diseases, identify their origins at an early stage, forecast developments more reliably, and make them usable for tailored prevention and therapy approaches in medical care (precision medicine/personalized medicine) [11,12,13,14,15,16,17,18,19].

Systems medicine is substantially dependent on innovative key technologies. These include, for example, high-throughput omics technologies, bioinformatics and medical informatics, artificial intelligence (AI), and big data analytics [20,21,22,23,24,25,26,27,28]. The SARS-CoV-2 pandemic also triggered a digitalization offensive in the healthcare sector; innovations were implemented much faster under the impact of the pandemic [29,30]. Since the German parliament passed a digital healthcare act (Digital-Versorgungs-Gesetz/DVG) in December 2019, which provides a new benefit entitlement for the use of digital health apps, Germany was well positioned to approve digital health applications in 2020 [31]. This makes Germany the first country in which digital health applications are approved as part of standard care and can be prescribed by a physician. Many nations, including Germany, have implemented mobile software applications (“apps”) as alarm systems to facilitate fast contact tracing and notification of potentially affected persons in the SARS-CoV-2 pandemic. In addition to this, the mass data collected on the digital platforms are an immense source of new insights [32]. Digital technologies can also complement or, in some cases, even reinforce the implementation of global health programs [32,33]. Systems medicine approaches are already helping to better understand the dynamics of SARS-CoV-2 spread and disease progression, and, for example, to identify appropriate drugs to treat SARS-CoV-2 through intelligent data analysis [34,35,36].

The main purpose of implementation research is to identify factors that influence the implementation process, e.g., at different levels (individuals, group of professionals, the innovation itself). They determine whether the implementation is successful or not [37,38]. It is not yet clear how systems medicine will develop in the future. Future research attempts to capture phenomena of the current situation from which future developments can be derived and predicted. The results are intended to support innovation processes [39]. When a linear forward projection of the current situation is not possible and uncertainties prevail, a systematic use of foresight methodologies can help to elicit opportunities and risks of new approaches. Future expectations and ideas should include all influencing factors, including the societal perspective. It is essential to actively deal with desired as well as undesired future trends. On this basis, impulses can be set, especially for political decisions [40,41]. So far, this process lacks the perspective of patients or the civil population towards the implementation of systems medicine approaches in healthcare. Existing studies on current and future challenges (e.g., ethical, legal, technical, medical, and societal issues) for the development of systems medicine primarily reflect expert perspectives [17,42,43,44,45,46,47,48,49,50]. Studies on attitudes of the civil population or of patients, for example, on digitalization, innovative technologies, and predictive tests, focus exclusively on such individual components [51,52,53,54,55].

The aim of this qualitative description study is to reveal the challenges of an implementation from the perspective of civil population and patient groups based on a future scenario of systems medicine-orientated healthcare. It is not an inventory of individual participants’ experiences, but a systematic and critical examination of their views on future trends. On this basis, possible facilitating and hindering factors are identified and a more detailed understanding is provided that may influence the future implementation success of systems medicine. From the qualitative findings, hypotheses and arguments are derived with respect to a possible implementation of systems medicine from the perspective of citizens and patients.

## 2. Materials and Methods

### 2.1. Design

The focus group interviews presented here are part of a qualitative research project using an exploratory sequential approach and mixed-methods design [56,57]. The overall aim of the mixed-methods design was to find factors that could potentially influence the implementation of systems medicine from diverse perspectives (citizens, patients, experts from science, economy, administration, politics, health and health-related stakeholders). The literature review (PubMed, Springerlink, Wiley Online Library, Livivo, search by hand) (Phase 1) on facilitators and barriers to systems medicine implementation did not yield robust findings. Results on systems medicine existed in the field of basic research and in expert debates on future healthcare. For further exploration, a qualitative approach with focus groups with citizens and a patient group was chosen (Phase 2), as well as face-to-face interviews with experts who had some contact with systems medicine (Phase 3). The aim was to find a variety of perspectives for idea aggregation and hypothesis generation to operationalize items for the Delphi process (Phase 4). In the Delphi process, arguments and hypotheses on potential influencing factors and chances of success for the implementation of systems medicine were evaluated and assessed by experts from medical care, politics, business, and science and research [10]. The results of Phase 2 are presented below.

This study is based on the methodological framework of qualitative description [58] and qualitative content analysis [59]. The qualitative design was reasonable for this study to explore key issues that might influence the implementation process [60]. This approach is appropriate for establishing the foundations to develop hypotheses and ideas [58,61]. To this end, focus groups are appropriate for eliciting attitudes, expectations, and emotional and social arguments in an interactive group process. The purpose of this study was to explore the perspectives of the general population and a patient group on systems medicine. Focus groups are a moderated, structured group process in which a thematic stimulus is presented as a starting point for discussion [62,63,64]. The focus group method has also become popular in technology forecasting, which assesses, among other things, the opportunities and risks of implementing (technical) innovations [65]. This could be transferred to the research question of the present study.

The study is reported following the recommendation of COnsolidated criteria for REporting Qualitative studies (COREQ) [66]. The study is registered in the German Clinical Trials Register (DRKS-ID: DRKS00008955).

### 2.2. Participant Recruitment and Sample

Between August 2015 and March 2016, citizens and a selected group of patients (people seeking advice and help who turned to an early diagnosis and treatment center for mental crisis (FETZ)) were recruited. The prerequisites for participation were a minimum age of 18 years, sufficient knowledge of German, and cognitive abilities to actively participate in a discussion group. Participation was voluntary; all participants provided signed informed consent. Knowledge about systems medicine was not required.

Citizens were made aware of our study “The medicine of the future” regionally in public places in Cologne (citizen service offices, University Hospital Cologne, adult education center) with a poster and via an announcement in a daily newspaper. The citizens represent a sample group of the general population. The members of the patient group were informed by their physician at the FETZ and asked whether they wanted to participate in the study. If they agreed, they were referred to the study management and contacted them independently. All participants received EUR 30 as compensation for taking part in the study.

A purposive sampling was used with the aim of variance maximization with respect to age and gender [67].

### 2.3. Conducting the Focus Groups and Data Collection

A vision of systems medicine implementations in everyday life was presented in the form of a narrative case vignette as a basis for a shared understanding of the topic and to stimulate discussion in the focus groups (see Box 1) [68,69,70].

Box 1 **Stimulus as a starting point for discussion in the focus groups: A Vision of Systems Medicine Healthcare in the Future.**

Mr. Miller is in his early 30s, and works as an assistant store manager in retail. He feels healthy and enjoys life to the fullest. He is invited by his GP to a “30+ health checkup.”

Mr. Miller attends the appointment. The following examinations and procedures are performed:

- Answering a health questionnaire;
- Medical examinations (physical exam, lab values: blood, urine; diagnostic images: ECG, ultrasound, MRI);
- Genetic analysis (DNA analysis);
- Information on personal lifestyle (smoking status, diet, fitness, place of residence, working conditions).

All the data are linked in an intelligent medical data model and analyzed using computer software. As a result, Mr. Miller receives his health profile and a prediction of his individual risk of disease. Mr. Miller has high blood pressure and is slightly overweight. There is a 90% chance of suffering a heart attack and depression in the next 15 years.

Based on the data, the system draws up a tailored prevention and treatment plan for Mr. Miller. The GP then recommends a change in diet, regular exercise, training to reduce stress, and participation in specific early detection measures for cardiovascular and mental illness. In addition, the GP prescribes a medication to lower blood pressure that is tailored to Mr. Miller’s genetic profile. The medication has minimal side effects, is low-dose, and specifically targets his high blood pressure.

The GP prescribes Mr. Miller a smartwatch for monitoring. Adherence to the GP’s recommendations and vital data are monitored continuously. These include:

- Nutritional status, fitness level, sleep patterns, and stress levels;
- Blood pressure and pulse.

Every day, Mr. Miller takes a selfie (self-portrait) with his smartphone. A special software program analyzes his facial expression and physical features in the photo. These are intended to reveal changes in his mental health.

He is reminded to take his tablet to lower his blood pressure by his smartwatch. Before he has taken his last tablet, Mr. Miller receives an electronic prescription, which he sends online to his pharmacy.

All data are collected, stored and evaluated in his electronic health record. Via his smartwatch, Mr. Miller regularly receives tips and recommendations for improved therapy adherence and is motivated to optimize his lifestyle. A feedback system ensures that his attending physician is informed and can intervene immediately if medically necessary. His next appointment with his physician is steered via his health profile. His medical checkups are scheduled electronically via his private and business diaries to ensure continuous or early treatment.

Because Mr. Miller consistently and successfully participates in the recommended monitoring program, he receives an annual premium payment from his health insurance company as a reward.

A semi-structured guide was systematically developed to conduct the focus groups [71]:

1. What are the perceived opportunities and risks of systems medicine based on the case vignette?
2. Which arguments are used for or against the development and use of systems medicine?
3. Which requirements and general conditions can be expected for the future development of systems medicine?

The interview guide and stimulus (case vignette, see Box 1) were developed by extracting key aspects and themes in the context of systems medicine from the previously conducted literature review (Phase 1). The semi-structured guide included open-ended questions that could be used in a flexible order. The development process of the semi-structured guide and the stimulus (case vignette, see Box 1) were validated in a discursive process in a multidisciplinary research team. In a pretest, both instruments were tested for functionality and comprehensibility, and the process was tested in simulated focus group interviews. The techniques of discussion management and co-moderation were trained.

The focus groups took place in the rooms of University Hospital Cologne. They were neutrally guided by a moderator (first author) and supported by a co-moderator. The co-moderator’s task was to note down on moderation cards, in a condensed and precise manner, the opportunities and risks perceived for systems medicine in the discussion process of the participants. At the end of the first discussion sequence, the moderation cards were clustered according to opportunities and risks, visualized for the participants, and validated communicatively (member check). The visualization served as an orientation aid in the further course of the discussion. At the end of each focus group session, the communication situation and atmosphere were reflected on by the moderator and co-moderator and documented in a protocol. The course of the focus groups was digitally recorded. The conversation content was transcribed according the simple transcription rules by Dresing et al. (2015) [72]. At least two focus groups were planned in each sample group until thematic saturation was achieved, i.e., no new categories emerged from the data [73,74].

### 2.4. Data Analysis

The data analysis was rule-guided and systematic, following the qualitative content analysis according to Mayring (2015). The categories were developed in a deductive–inductive manner [75]. The evaluation process was quality-assured by means of peer debriefing using an intra- and intercoder analysis to validate reliability [75,76].

Initially, the first author coded the transcripts. The main categories were derived deductively from the interview guide; further categories were inductively added and differentiated during the process. After about 25% of the coding process had been completed, the category system developed and the application of the categories to the data were validated by the second author, and ambiguities were discussed together. After a consensus had been reached in this way, the text material was coded again by the first author on this basis. Transcripts were categorized and analyzed using the MAXQDA 12 program (VERBI GmbH, Berlin, Germany). Results and interpretations of the in-depth analysis were discursively reviewed and consented by an independent multidisciplinary research team (from medicine, health science, and health economics).

## 3. Results

### 3.1. Participation in the Study

A total of 62 citizens and 13 patients agreed to participate in the study. Of these, 32 persons attended one of the four focus group sessions. Partial over-recruitment allowed for appropriate group size (five to six participants) with the desired maximization of variance (in regard to age and gender distribution) in each session.

Four focus group sessions were conducted with participants from the general population (n = 22; m = 9, f = 13, age = 24–78 (mean 47.6 (SD ±15.2); median 47.5)). Two focus group sessions were conducted with patients (n = 10; m = 9, f = 1, age = 18-35 (mean 27.5 (SD ±5.8); median 27.0)). Young males predominated in the patient group. The majority of FETZ patients seeking advice and support correspond to this population. The general population group also included participants who were not smartphone users (n = 3; m = 1, f = 2, age ≥ 65). Experience with mobile digital health applications was reported by 53.1% of the participants (n = 17; citizens n = 10 (45.5%), patients n = 7 (70.0%)). A total of 46.9% of the participants (n = 15; citizens n = 12 (54.5%), patients n = 3 (30.0%)), 21.9% (n = 7; citizens n = 5 (22.7%), patients n = 2 (20.0%) were open to using digital health apps (Table 1).

Key findings from the focus group interviews are presented below.

### 3.2. Future Image of Systems Medicine-Oriented Healthcare

The participants were able to get engaged and were interested in mentally playing out such a science fiction scenario for the use of systems medicine. The presented story (see Box 1) spontaneously triggered a variety of negative feelings and fears of uncontrollable developments in all focus groups.

*Well, I find it more concerning than progressive, really—a little horror scenario.*

(FG 6 P: 121–121)

(New) technologies were perceived as dominant and emotionally burdensome: Technology dominates and controls the body and mind.

*You become a slave to your own health because everything is all about technology.*

(FG 1 A: 80–80)

The direct link between health and technology is perceived as de-individualizing. Under such conditions, man would only be there to be a “healthy” labor force.

*But on the other hand, it also looks more like that the human being is simply a resource to be kept alive and which has to be trimmed to keep themselves healthy so that so they can work.*

(FG 5 P: 151–151)

Every individual in society would be completely screened and lose their privacy. Man would be monitored and would lead a life directed by others.

*Essentially, the idea of the “transparent patient” (someone who lost privacy) also occurred to me.*

(FG 1 A: 76–76)

*Monitored. A life determined and controlled by others—and both together in the overall view then—when I read this text.*

(FG3 A: 140–140)

The development of systems medicine disenfranchises people; it would take away their personal responsibility for their health.

*The personal responsibility, that’s what I’m missing here, TOO. And I think that should be trained much more, instead of being taken away.*

(FG 2 A: 137–137)

Some participants remarked that the awareness of one’s own body would get lost.

*And I’m just very afraid that every person would lose the basic trust in his/her body, because s/he then delegates everything to the technology and doesn’t really even listen to her/himself.*

(FG 1 A: 80–80)

Participants were concerned about the development of key technologies to predict diseases. People at risk could be stigmatized and discriminated against in society because of individual predispositions or the prediction of disease events (individual risk profiling).

*And then at some point, they’ll say: Okay, we’ll investigate that. But don’t have any children now, they would just be a burden on our society.*

(FG4 A: 111–111)

Fear of cyber-crime and data misuse was present in every group. This ranged from misuse scenarios such as unauthorized reading of and spying on personal health data, to manipulation and unwanted use of sensitive health-related data.

*Thus, the likelihood of such information packages being misused to the detriment of the patient in such a development, by whatever party, is much greater than the benefit.*

(FG 6 P 160314_0020: 135–135)

### 3.3. Arguments for and Against

Table 2 lists key arguments for and against the future development of systems medicine.

#### 3.3.1. Digitalization and the Use of Key Technologies

Similar viewpoints on digitalization and the use of key technologies in the context of systems medicine were addressed in all focus group sessions. Most participants perceived digital applications (e.g., e-prescriptions, electronic health records, digital reminder functions, real-time monitoring in everyday life) in medical care as a low-threshold technical solution. In their view, they offer advantages in emergency situations and/or in medical treatment. This makes it possible to comprehensively exchange health-related information in the care process across institutions and sectors. It was noted that digitalization enables physician–patient communication that is independent of time and place. The participants expected technical solutions to help to better coordinate consultation appointments, save travel and waiting times, and reduce healthcare costs. Data protection and security were rated as insufficient. Digitized health information might not be sufficiently protected from unauthorized access. Most focus group participants saw real-time monitoring as beneficial in the everyday lives of (chronically) ill people and seniors. Continuous data transmission and analysis of vital data (telemedical application via smartphone and sensors) would make it possible to detect decompensations or acute events at an early stage and even save lives in an emergency. On the other hand, they perceived the continuous occasion-independent measurement of vital data as a burden; permanent monitoring in everyday life would result in a loss of quality of life. The knowledge to adequately assess one’s own health would no longer be trained or would be lost; users could misinterpret their vital data.

Some participants saw the use of innovative techniques and AI for processing large health-related data sets as an opportunity for the medical process. They believed that the physician benefits from the use of such solutions (e.g., AI as an element of a clinical decision support system (CDSS)) in the medical decision-making process. A positive aspect is that patients’ disease risks can be identified at an early stage, precise diagnoses can be made, and the course of the disease and individual therapy success can be reliably predicted. Individual risk profiling was regarded as an opportunity. In this way, one could influence one’s own health and the course of the disease. In turn, they argued that the human art of healing, empathy, and personal communication between physicians and patients during the treatment process would be lost if people were reduced to data alone. Most of those discussants strongly believed that an individual risk assessment and definitive knowledge of one’s risk of disease would be a major emotional burden. They believed that knowing one’s prognoses and the practice of predicting disease risks would result in a “self-fulfilling prophecy,” as it would both consciously and sub-consciously influence the person’s behavior so the prediction would come to pass.

#### 3.3.2. Openness to/Willingness to Use a Systems Medicine Approach

Two of the 32 focus group participants indicated that they could envision participating in a systems medicine approach; they saw early detection of disease as a good opportunity for effective health promotion and prevention. Most of the focus group participants were open to this on certain conditions; some would consider using systems medicine for their own health if they became severely or (chronically) ill. For some participants, a balance between costs and benefits would be a necessary condition for use. Two participants rejected the idea of using systems medicine themselves.

The majority of the participants were open to sharing their anonymized health data for research purposes in systems medicine. They expected this to provide to new insights into the causes of diseases and the development of targeted therapies. Two of the persons from the general population group declined to make their data available for research purposes.

### 3.4. Ideas and Discourse on Prospects for Action

Table 3 summarizes the key ideas and discourses on the requirements and conditions for the potential implementation of systems medicine.

#### 3.4.1. Understanding of Systems Medicine among the General Population

In all the focus groups, knowledge about systems medicine, digital health literacy, strengthening of decision-making sovereignty, and skills in dealing with key technologies were regarded as basic prerequisites for the implementation of systems medicine. The participants believed that new occupational fields and the promotion of knowledge in schools (e.g., digital health literacy) could improve understanding among the general population.

#### 3.4.2. Financing of Systems Medicine-Oriented Healthcare Services

Different financing strategies for systems medicine services were debated. Most participants wanted solidarity-based financing to be ensured. For some, funding from taxpayers’ money or third-party funding from the private sector (manufacturers of drugs or medical devices) was conceivable.

#### 3.4.3. Use of Systems Medicine-Oriented Care Approaches in the Solidarity System

Health insurance companies could offer their policyholders incentives to make use of systems medicine services. This could be done in two ways: Policyholders could either be rewarded with bonus programs or sanctioned via higher health insurance premiums. Most participants were concerned that health insurance companies may require mandatory participation in systems medicine-oriented healthcare. In three focus groups, the willingness to pay co-payments and the agreement to cover costs were discussed. The opinions of the general population on these topics differed from those of the patient group. Many participants in the general population group (discussion topic in two of four FGs) tended to reject co-payment. It was expected that in the solidarity system, if the benefit is proven, the costs would be covered by the health insurance companies. Nevertheless, co-payment was conceivable in the case of voluntary participation in systems medicine-oriented services. In the patient group (discussion topic in one of two FGs), there was a conditional willingness to make co-payments. The premise would be the proven benefits of systems medicine and the exemption of socioeconomically disadvantaged groups from co-payment.

#### 3.4.4. Data Protection and Security When Dealing with Big Data

In the context of systems medicine, focus group participants lacked confidence in existing structures and security standards for processing sensitive health data. Protection against unauthorized access, cyberattacks, and data misuse were discussed critically; as an external condition, the technical prerequisites for comprehensive data protection would first have to be established.

#### 3.4.5. Normative Implications

Ethical and legal aspects were discussed intensively in the focus groups. Systems medicine would allow deep insights into a person’s state of health, personality, and lifestyle. The participants feared that values such as freedom, (informational) self-determination, equal opportunities, responsibility for one’s own health, decision-making sovereignty, and the solidarity system would be at risk. It would have to be clarified how a systems medicine service could be made available to every individual in society and how it could be ensured that personal rights (e.g., protection against discrimination, informational self-determination) are not violated in the process. From the perspective of the discussants, it is necessary to have a social discourse on sustainable and appropriate strategies.

## 4. Discussion

The aim of this study was to create an initial knowledge base on the potential opportunities and risks as well as future expectations regarding the implementation of systems medicine from the perspective of citizens and patients. They were asked about their expectations for future healthcare with systems medicine: Certain aspects were seen as positive, but overall their perspective was dominated by consideration of the risks. Uncertainty existed regarding the high level of technification and the idea of having complex health-related data analyzed for standard healthcare. The latter could be used to derive quite precise risk-prediction models for disease events and trajectories for each person. There were normative and ethical concerns, e.g., regarding the undesirable use of data and the restrictions of fundamental rights.

### 4.1. A Vision of Systems Medicine-Oriented Healthcare in the Future

The introduction of the case vignette (see Box 1) led to defensive reactions and emotional skepticism in the discussion groups. The use of systems medicine-oriented techniques was associated with a takeover of control over people. The confrontation of one’s own physical, emotional, or mental states with a sensually inaccessible and incomprehensible (digital) technology was perceived as threatening. People obviously react sensitively to technology (machines, computers) whenever it intrudes into areas that affect their self-image as human beings. It may be possible to explain the focus group participants’ response using the human–machine interaction model. Although this term traditionally referred exclusively to industrial machines (as a substitute for body power), it is now also applied to new technologies such as computers, digital systems, and robotics [77,78,79]. Körner et al. (2019) investigated stressors associated with the introduction of highly automated technologies in the manufacturing industry. When employees are overwhelmed with the use of innovative technologies, this triggers work-related stress, which, among other factors, negatively affects psychosocial health [80]. Weidemann and Rußwinkel (2021) described what happens when problems occur in the interaction between humans and robots. When robotic systems display erroneous behavior, it triggers counterproductive emotional states in users, such as frustration, feelings of inferiority to technology, and loss of control [81]. As for a well-functioning human–machine interaction, the concerns, expectations, and capabilities of users regarding new key technologies should be considered when implementing systems medicine. Heßler (2019) assumed that feelings of (perceived) control and human superiority have been essential for the acceptance of innovative key technologies so far [82].

### 4.2. Arguments for and Against

The development of systems medicine is linked, among other things, to digital transformation and the establishment of digital health applications [24,43]. The participants in the focus group were generally open to (smart) technologies and a cross-linked healthcare system. Resources in the healthcare system could be saved, and better networking could ensure optimal coordination and communication between the treatment team and the patient. (Chronically) ill patients and seniors could benefit from digital solutions for effective therapy and escalation management. This finding was confirmed in a survey by McKinsey (2020) on the attitudes of Germans toward digital healthcare services. Two-thirds stated they had a positive attitude toward digital health services. The willingness to use them had increased significantly in all age groups since the beginning of the COVID-19 pandemic [52]. Despite the open-mindedness, there were many counterarguments and concerns in the focus groups. The participants tended to associate continuous monitoring of health values in everyday life with a loss of quality of life and well-being. They were concerned about adequate data protection and control options regarding the security of sensitive health data. In line with the present study, an analysis of the “TechnikRadar” 2018 [83] and 2019 [84] confirms on the one hand the largely positive attitude toward technical developments and digitization among the German population, but also fears on the other. The main concerns are the susceptibility of infrastructures to cyber-criminality, failure or manipulation, and the possible loss of control over one’s own data [53].

The findings of this study show that increasing digital connectedness and experience with smart technology in various areas of life have triggered a process of change within society. Nevertheless, progress in setting up the telematics infrastructure and introducing digital health applications in Germany has been slow in recent years. The Act to Modernize the National Health Insurance System of 14 November 2003 already legally stipulated the introduction of the electronic health card (eHC) on 1 January 2006 (Section 291a of the German Social Code, Book V (SGB V)). This was to enable digital data processing and access to an emergency data set, medical information in an electronic health record (EHR), storage of e-prescriptions, and drug therapy safety review data (eMedication Plan). Compared to other European countries, Germany was in the middle of the field in 2016 when it came to setting up the EHR. The successful implementation of a national e-health strategy requires modular and pragmatic approaches and good communication strategy by policymakers regarding the benefits for users (patients and service providers) [85]. Since the SARS-CoV-2 pandemic, it seems as if the digitization of the German healthcare system has finally taken off [29,30]. A mandatory stepwise rollout of the EHR from 2021 and the mandatory use of the e-prescription from January 2022 was decreed by the Patient Data Protection Act (PDSG) [86]. Other COVID-19-induced IT-developments, such as the Corona-WarnApp, which is a track-and-trace application [87], and the German Electronic Reporting and Information System for Infection Control (DEMIS), a nationwide secure electronic reporting and information processing system for cases of positive SARS-CoV2 pathogen detection [88], are examples of a cautious digital transformation.

Another field of development of systems medicine is big data approaches and the application of artificial intelligence (AI) for highly individualized medical care [28,89,90]. This approach was intensively discussed by the participants regarding its possible consequences for health care practice. Although the opportunities were also perceived, the risk-benefit ratio tended to be assessed negatively. The possibility of using systems medicine integration to gain insight into a person’s health condition and lifestyle unsettled participants. The predictions of disease development and the grading of individual risk from “healthy” to “risk of disease” to “manifest disease” were perceived as burdensome; with such knowledge people would fall into a kind of “self-fulfilling prophecy.” These concerns, particularly predictive risk assessment (individual risk profile) in standard care, appeared to impact reluctance to use systems medicine approaches. Wegwarth et al. (2019) examined attitudes and potential willingness to use predictive epigenetic testing for cancer risk (breast, ovarian, cervical, and endometrial cancer) among women in five European countries (Czech Republic, Germany, the UK, Italy, and Sweden) in a cross-sectional online survey. More than half of the women said they wanted to know their individual 10-year risk. They saw a potential benefit. Nevertheless, they feared—like the focus group participants in this study—that knowledge about disease risks would lead to unnecessary worry and thus have a lasting impact on their own lifestyle and quality of life. Women with low knowledge about the testing procedures generally estimated higher harm than potential benefit [51]. In a further qualitative study, a potential user group was asked about the possible use of big data approaches for individual mental health risk prediction. Half of the respondents could envision using it, whereas more than a third said they would reject it [54]. By comparison, the studies by Wegwarth et al. (2019) and Mantell et al. (2021) showed that respondents were more open-minded regarding the use of systems medicine approaches than in the present study. However, the comparison groups in the literature are each limited to a single field of application. Apparently, the quantity, variety, and interdependence of the components of systems medicine unsettled participants.

In contrast to their reluctance regarding the use of systems medicine in healthcare, most discussants were willing to share anonymized personal health-related data for research. This willingness was also supported by studies by Brall et al. (2021) and Richter et al. (2021). There is a positive attitude toward the use and security of anonymized health data for research and development. The public seems to trust researchers to use donated data to investigate causes of how diseases develop and spread; they are convinced that the knowledge gained will enable the development of innovative prevention, diagnosis, and treatment strategies [91,92].

### 4.3. Perspectives for Implemention

From the perspective of the discussants, competencies in dealing with digital technologies and the development of a basic understanding of systems medicine are considered essential. Education initiatives are needed for individuals to understand and correctly interpret systems medicine and to apply it to their own health-related decisions and actions. The introduction of systems medicine-oriented technologies and the use of digital applications in the healthcare system increasingly require digital health literacy. This includes the ability to use digital technologies in a self-determined way for the purpose of maintaining, recovering, or improving health [93]. The concept of digital health literacy is seen as a key element in connecting digital and health aspects [94,95]. Knowledge of systems medicine, a positive attitude toward its possibilities, and skills in using key technologies in the context of systems medicine approaches could improve public acceptance. As Damschroder et al. (2009) state, the triad of knowledge, a positive attitude, and hands-on experience are said to make a person more likely to accept and use innovations [96].

The focus group participants expected and wished for the further development of systems medicine to be supported by the solidarity of the insured and that reimbursement for the required services would be ensured by the statutory health insurance (SHI). The SHI is a central element of the German healthcare system. Every person covered by the SHI has the same entitlement to benefits under the provisions of Volume 5 of the German Social Code (SGB V) regardless of his or her risks or income [97]. To what extent systems medicine can find its way into the existing SHI benefits law is still open. The current legal framework hardly allows systems medicine to qualify as standard treatment [98]. The extension of benefit law to algorithmically generated risk profiles in healthy individuals is seen as a particular challenge because SHI benefit law is currently linked to defined diseases (ICD-10) [47].

The participants in the focus groups were particularly clear about their needs regarding data privacy, the guarantee of basic individual rights, and compliance with fundamental ethical principles. Among other things, the handling of highly sensitive health data and the fundamental right to (informational) self-determination were named as potentially endangered values. The use of systems medicine requires a regulatory framework (e.g., regarding cloud computing, protection of privacy, right not to know, voluntariness) and an ethical justification (e.g., autonomy, care, justice). The German legal system either lacks standards and legal regulations for this or they are not fully applicable in the context of systems medicine [47,50,98]. A broad implementation of systems medicine can only succeed if equality of opportunity, protection against discrimination, solidarity, and individual self-determination are guaranteed. In Germany, Der Deutsche Ethikrat (the German Ethics Council) and several other public and scientific institutions are promoting a discourse on the ethical and cultural issues on biomedicine, big data, robotics, and artificial intelligence (AI), which can be classified as important precursors of systems medicine.

### 4.4. Limitations

In the present study, some limitations regarding the composition of the sample and the qualitative research approach must be considered. First, the participants in the focus groups were persons from the city of Cologne and nearby municipalities. This implies an over-representation of the urban population. Secondly, the number of patients was rather small in relation to the general population; furthermore, young men were over-represented in this group. However, this potential patient user group broadened the perspectives; they had a hypothetical interest in identifying and treating mental health disorders as early as possible. Third, the potential study participants were selected (purposive selection) based on certain characteristics of interest to the study; as a basic requirement they needed to be able to actively participate in a discussion group. Therefore, there was a selection bias because citizens who were not actively participating in or interested in health services were not included. Qualitative research in principle does not aim at making empirically generalizable statements about the participants, but at collecting contrasting views. Fourth, the perspectives for action discussed were not based on the empirical knowledge of the participants regarding systems medicine approaches, but on their associations, ideas, expectations for the future, and subjective perception of a constructed future scenario. The participants’ contributions provided important input for the discussion on the development of implementation strategies.

## 5. Conclusions

A broad application of systems medicine in healthcare is currently still a utopia. Systems medicine is highly complex and therefore difficult to grasp. Nevertheless, dynamic (bio)medical technological developments and digital transformation are constantly opening up new ways to integrate systems medicine approaches into healthcare.

Weighing the opportunities and risks for the development and implementation of systems medicine is likely to be a major political and social challenge in the future. Civilians should be made aware of and prepared for developments such as the use of big data-driven AI procedures for predictive risk assessment and profiling (e-health literacy). This includes self-determined participation in systems medicine-oriented services and the individual ability to deal with potential risks of systems medicine. In addition to information about systems medicine and its potential benefits, perceived concerns and needs must be adequately addressed and considered in the development of appropriate health policy implementation strategies. There are significant acceptance risks on the part of citizens that must be overcome. To this end, suitable regulatory, legal, and economic framework conditions must be established, e.g., on the issues of data protection, responsibility, transparency, and discrimination. Ultimately, it will depend on everyone’s acceptance whether systems medicine can step out of its niche and take a meaningful place in the healthcare system of the future.

## Figures and Tables

**Table 1:** Characteristics of study participants—focus group interviews.

		Total		Citizens		Patients ^1^	
Participants	n	32		22		10	
Focus group rounds	n	6		4		2	
Gender							
Male	n (%)	18	56.3%	9	40.9%	9	90.0%
Female	n (%)	14	43.8%	13	59.1%	1	10.0%
Age group (years)							
18 to 25	n (%)	5	15.6%	1	4.5%	4	40.0%
26 to 40	n (%)	11	34.4%	5	22.7%	6	60.0%
41 to 55	n (%)	10	31.3%	10	45.5%	0	0.0%
56 to 70	n (%)	4	12.5%	4	18.2%	0	0.0%
≥71	n (%)	2	6.3%	2	9.1%	0	0.0%
Age (years)							
	Mean ± SD	41.3	± 16.0	47.6	± 15.2	27.5	± 5.8
	Median	38.0		47.5		27.0	
	Min.	18		24		18	
	Max.	78		78		35	
Smartphone user							
Yes	n (%)	29	90.6%	19	86.4%	10	100.0%
No	n (%)	3	9.4%	3	13.6%	0	0.0%
Experience with digital health (apps, wearables, and sensors)							
Yes	n (%)	17	53.1%	10	45.5%	7	70.0%
No	n (%)	15	46.9%	12	54.5%	3	30.0%
Rejection	n (%)	8	25.0%	7	31.8%	1	10.0%
Application conceivable	n (%)	7	21.9%	5	22.7%	2	20.0%

^1^ Patients with early onset of a mental disorder.

**Table 2:** Key results of arguments for and against—future development of systems medicine.

**Digitalization and Use of Key Technologies**			
**Topic**	**Pro**	**Cons**	**Selected Quotes**
**Electronic prescription**	Complete documentation of medication Saving travel and waiting times		*What I found quite good is that if you have now taken your last blood pressure tablet, you get an electronic prescription directly. I think that’s great. It saves me a trip to the doctor.* (FG 3 A: 150–150)
**Electronic health record**	Availability across facilities and sectors Access to health-related information in emergency situations and for medical treatment Avoidance of duplicate examination Cost reduction	Unauthorized access to sensitive health data Misuse of sensitive health data	*But I would just also see it in context with the specialists; that all the physicians I go to get the same information.* (FG 1 A: 150–150) *Well, the advantage of storing it centrally is*… *if I’m taken to hospital, for example, am unconscious… then they know straight away: Okay, he’s allergic to penicillin and other antibiotics.* (FG 5 P: 189–189) *The whole thing goes hand-in-hand with a reduction of costs, because duplicate examinations are avoided or that the patient sees numerous physicians.* (FG 4 A: 80–80)
**Digital reminder function**	Reminder to take medication Coordination of examination appointments Low-threshold technical solution	Monitoring and control via access authorization for third parties to one’s own diary	*In terms of assistance…the whole tablet thing, making appointments—those are the kinds of things where I would say, these things are very low-threshold and they’re usable.* (FG 3 A: 159–159) *Well, that means they’re monitoring everything—they can even look at your diary, for example. And I think sometimes that’s going a bit too far.* (FG 6 P: 112–112)
**Real-time monitoring in everyday life**	Digital physician–patient communication anytime, anywhere Early warning system before decompensation or acute events; especially for (chronically) ill patients/seniors Support of self-management for chronically ill patients Low-threshold access in rural healthcare structures	Loss of quality of life Fear that a layperson may misinterpret these vital parameters Lack of knowledge to adequately assess one’s own health	*However, if you need to talk, you could also connect this smartwatch with telemedicine, so that you can at least call the physician* via *it and then you have this contact with the physician.* (FG4 A 151111_0012: 313–313) *…, so, this acute situation, when you feel really bad, that you can just “click” and the emergency ambulance comes.* (FG 2 A: 176–176) *And you would see that very early on, if the person has diabetes and… derails, then you could counter that…* (FG 5 P: 302–302) *So when I think about patients who receive chemotherapy, it makes sense to have such a close control…* (FG 1 A: 96–96) *For rural physicians…, for patients who are now also older, who have no possibility to go to the physician and who now have complaints.* (FG 3 A: 171–171) *Yes. Exactly, if it comes to a permanent use of the watch and everybody gets every week “Don’t get so upset” or so on the watch and “Otherwise this disease will come,” of course a big part of the joy of life is lost.* (FG 5 P: 151–151) *So once the value is up a little bit, depending on what type they are, then that person immediately rushes to the physician, even though it may not be that acute.* (FG 2 A: 180–180)
**Big data analytics**	Optimal and individually tailored therapy Early identification of disease risks Precise diagnoses Individual risk profiling as an opportunity to influence a patient’s health or the course of the disease Supports the medical decision-making processes	Prediction of disease risks could lead to so-called self-fulfilling prophecy The concrete knowledge of one’s own disease risk/prognosis (individual risk profiling) is emotionally stressful Concerns that empathy and communication will be lost in the treatment process Reduction of people to their data Lack of trust in AI-based analysis methods for diagnoses and prognoses Lack of data and risk literacy	*Selection of the drug based on the data. I think that’s good the drug has low side effects, is low dose and is specific against hypertension so if on the basis of this data evaluation, this drug is also is determined in the same way.* (FG 1 A: 70–70) *I don’t know what you can find out with this, but probably there will be more and more diseases, which you can perhaps determine relatively precisely in advance, the better this gene analysis becomes I would do it, too.* (FG 6 P: 145–145) *So, based on this scenario, I think that an individual therapy is possible for the patient, which is tailored to the patient.... This also has a prophylactic effect, because you can say in advance, what is the probability of a disease?* (FG 4 A: 80–80) *Yes, I also think that the physician is supported more by the fact that he gets more data from the person and can then also assess them more quickly.* (FG 6 P: 298–298) *It can also be that through the diagnosis, that someone says, “you will get cancer or depression with 90% probability,” that the person still talks himself into it and virtually prophesies it himself.* (FG 5 P: 168–168) *So I think that will be based even more on data and less on clinical view and empathy, so looking at the patient, just listening—because you make the big diagnosis in a quarter-hour conversation—in that time you’ve figured out half, at least, oh, 80, 90 percent of the diagnosis.* (FG 2 A 151015_0021: 259–259) *I don’t know how they calculate that … then I would worry a lot. And the depression would probably be there much faster. So I wouldn’t like that so much.* (FG 6 FETZ 160314_0020: 131–131)
**Openness to/Willingness to Use Systems Medicine Techniques**			
**Topic**	**(Conditional) Acceptance**	**Rejection**	**Selected Quotes**
**Utilization for own health (e.g., big data analytics,** **individual risk profiling)**	Early diagnosis of diseases for effective health promotion and prevention As support for targeted prevention and treatment strategies for those with serious or chronic illnesses Coherent relationship between effort and benefit	Lack of conviction	*Yes, I would also monitor from a healthy state, if that would help to FIND any serious diseases in advance and then also prevent them afterwards perhaps or counteract—why not?* (FG 6 P 160314_0020: 165–165) *Well, I could imagine, if I have an acute illness, for example, if I am now a cancer patient and thereby the future patients could benefit from it.* (FG 5 P 151214_0019: 165–165) *Okay, with me it would be diabetes. Definitely. I’d be open to that.* (FG 2 A 151015_0021: 166–166) *Okay. So, I can imagine that very well, if the risk-benefit ratio is appropriate and the effort is right.* (FG 1 A 150928_0007: 86–86) *But for me it would also depend on the initial diagnosis. So, with a 90 percent risk of getting depression or a heart attack in the next 15 years, I’d accept quite a lot (laughs) already.* (FG 5 P 151214_0019: 149–149) *Well, I can’t imagine that at all in that sense, even if I could influence it. So, I think I’m perhaps still very bourgeois and old school.* (FG4 A 151111_0012: 94–94)
**Sharing anonymized health data (big data) for research**	High potential for new insights	Rejection	*Yes, I’d do it. (FG 2 A 151015_0021: 195–195)* *Same for me. I wouldn’t have any problem with it.* (FG 6 P 160314_0020: 182–182) *Yes, if there was some disease where I could help to develop something, then also, but otherwise it would have to have then already really relevance.* (FG 1 A 150928_0007: 122–122) *I wouldn’t do it.* (FG4 A 151111_0012: 163–163)

**Table 3:** Key results on ideas and discourse on areas for action—future development opportunities for systems medicine.

Requirements and Conditions for Implementation			
Topic	Area of Action	Ideas/Discourse	Selected Quotes
**Understanding of systems medicine in the general population**	Digital health literacy Decision-making sovereignty and competencies in dealing with key technologies	New professional domains Knowledge promotion in schools (e-health literacy)	*Maybe there is a person who is already there with the physician, who can explain it to you. That maybe there’s an extra room where you can talk to someone and get the information.* (FG 2 A 151015_0021: 316–316) *Trained staff that knows about this product and can explain it to me; if I have a chronic condition, I might have to use a device.* (FG3 A 151029_0023: 331–331) *Well, the health insurance companies should inform their members about new developments.* (FG 6 P 160314_0020: 290–290)
**Financing of systems medicine-** **oriented healthcare services**	Financial strategy	Financing from state/tax money Financing by the private sector Solidarity-based financing	*First of all, a huge amount of money has to come in to get the system up and running. The state would have to pay for that.* (FG 6 P 160314_0020: 208–208) *I’d say the pharmaceuticals industry could do it, too.* (FG4 A 151111_0012: 189–189) *Well, for me it seems pretty obvious that the health insurance companies SHOULD cover a large part of the costs because they should have had some thoughts in advance about how useful the whole thing is.* (FG 1 A 150928_0007: 130–130)
**Use of systems medicine-** **oriented care approaches in the solidarity system**	Incentives for taking individual responsibility for health	Incentives via health insurance premium (bonus) Sanctions via health insurance premium (malus)	*They need to pay a higher health insurance premium. That’s the only way they’ll get people on board.* (FG4 A 151111_0012: 325–325) *I think that’s okay if those who make a little more effort and do more sports and also do a little bit for their health, that they just get a bonus on top of that. Maybe several bonus levels.* (FG 6 P 160314_0020: 213–213) *I don’t like the idea of the bonus system either. I think it gives wrong incentives to always choose the health insurance companies that give more bonus. And then more and more sick people are pushed into insurance companies that offer substandard coverage.* (FG 2 A 151015_0021: 381–381) *But I don’t think that’s good if you then increase the premium just because you say I don’t want that.* (FG 2 A 151015_0021: 403–403) *It may be, of course, if in 10, 20 years certain diseases, for whatever reason, are now accumulating, that in the specific case they may then sanction unwilling patients in some way.* (FG 6 P 160314_0020: 212–212)
	Willingness to pay and coverage of costs	Co-payment Freedom of choice Full cost coverage	*So, if that had a really big benefit, yes, I would pay for it; but I don’t want to be financially overburdened.* (FG 5 P 151214_0019: 382–382) *In my opinion, that is very individual. Everyone has to know for themselves how much they are worth to themselves (laughs), as far as health is concerned, let’s put it that way.* (FG 1 A 150928_0007: 145–145) *Well, I wouldn’t want to pay anything on top of that either. I wouldn’t have any insight into that either.* (FG 2 A 151015_0021: 383–383)
**Data protection and security for the management of big data**	Data protection and security	IT infrastructures with the highest security standards Cyberattacks and data misuse Access control Cloud solutions	*The only thing that is certain is that nothing is secure. You may be able to secure that temporarily somehow, but in the long run, there is no system that you can’t crack.* (FG 2 A 151015_0021: 193–193) *But otherwise, I think it’s not a bad idea to say: This is somehow stored with the GP and he ADMINISTRATES it, so to speak, and then passes it on. That would be the safest method for me, safer than that it wanders around somewhere, I don’t know, yes, in a cloud or something*. (FG 1 A 150928_0007: 175–175) *The way I see it, the greatest benefit also comes with the greatest risk. I mean, if it’s stored centrally, that is where it can be most beneficial, but that’s also the situation with the most risk... And it’s just not possible to make something like that 100 percent secure.* (FG 5 P 151214_0019: 271–271)
**Normative** **implications**	Autonomy, privacy, and (informational) self-determination	Responsibility for one’s own health Decision-making sovereignty for the utilization Right to self-determination (constitutional right (within Germany)) to the “free development of the personality” Art. 2, Para 1, Basic Law of the Federal Republic of Germany [GG]) Informational self-determination (German Basic Law: General right of personality Art. 2, Para 1, GG/Art. 1 Para 1, GG) and control over one’s own data (Art. 8, EU Charter of Fundamental Rights)	*Everyone is responsible for themselves. And it depends on where that starts now. Whether it starts purely in prevention or whether it starts in therapy… And if someone doesn’t want that, that’s okay as well. It’s their decision, and their right*. (FG 1 A 150928_000702.07.2020: 147–147) *Well, I don’t think it would be at all acceptable to force it on people.* (FG 5 P 151214_0019: 281–281) *Exactly, people should be the masters of their data and the legislator must enforce this. Rigorously. Also, against others.* (FG 2 A 151015_0021: 264–264)
	Equal opportunities and discrimination	Exclusivity of systems medical services Discrimination against people with low income Endangering of personal rights	*Well, that’s where all the ethics come into play. One could also say that someone who has lived a healthy life before might have to pay less. But the question is: Does this exclude others? So, you will be confronted with many new problems. Then there is also the question of income: Does the person who earns more also have to pay more? So, I still see many, many question marks.* (FG 1 A 150928_0007: 152–152) *You are either left with the costs or you cannot take preventive action against your illness. And then I see the problem that it can also lead to exclusion in SOCIETY. You have the people who are, let’s say, motivated and also those who aren’t and do that.* (FG 1 A 150928_0007: 148–148) *Strictly speaking, that would be discrimination. That would be an infringement of personal rights.* (FG 6 P 160314_0020: 212–212)
	Welfare state principle	Legal right to healthcare Social consensus on sustainable and appropriate strategies for financing	*I just wanted to say, this is, after all, enshrined in the Basic Law and also in the Social Law—we all have a right to preventive healthcare, whether we’ve got INSURANCE or not.* (FG4 A 151111_0012: 330–330) *We need a consensus there. Because the individual citizen can’t carry the financial burden.* (FG3 A 151029_0023: 296–296)

## Data Availability

All the data relevant to the study are included in the article. Since complete transcripts of focus group interviews would potentially enable the identification of individuals, complete transcripts cannot be provided.
